# Dose-response association between device-measured physical activity and incident dementia: a prospective study from UK Biobank

**DOI:** 10.1186/s12916-021-02172-5

**Published:** 2021-12-02

**Authors:** Fanny Petermann-Rocha, Donald M. Lyall, Stuart R. Gray, Jason M. R. Gill, Naveed Sattar, Paul Welsh, Terence J. Quinn, William Stewart, Jill P. Pell, Frederick K. Ho, Carlos Celis-Morales

**Affiliations:** 1 Institute of Health and Wellbeing, University of Glasgow, Glasgow, G12 8TA UK; 2 British Heart Foundation Glasgow Cardiovascular Research Centre, Institute of Cardiovascular and Medical Sciences, University of Glasgow, Glasgow, G12 8TA UK; 3 Faculty of Medicine, Universidad Diego Portales, Santiago, Chile; 4 Institute of Neuroscience & Psychology, University of Glasgow, Glasgow, G12 8TA UK; 5 Centre of Exercise Physiology Research (CIFE), Universidad Mayor, Providencia, Chile; 6 Human Performance Lab, Education, Physical Activity and Health Research Unit, Universidad Católica del Maule, 3466706 Talca, Chile

**Keywords:** Accelerometer, Alzheimer’s disease, Dementia, Physical activity

## Abstract

**Background:**

Previous cohort studies have investigated the relationship between self-reported physical activity (PA) and dementia. Evidence from objective device-measured PA data is lacking. This study aimed to explore the association of device-measured PA with the risk of dementia incidence and common subtypes (Alzheimer’s disease [AD] and vascular dementia) using the UK Biobank study.

**Methods:**

84,854 participants (55.8% women), invited to participate in the device-measured PA between 2013 and 2015, were included in this prospective cohort study. Wrist accelerometers were used to measure light, moderate, vigorous, moderate-to-vigorous PA (MVPA) and total PA intensity and duration (MET/min/week). Incident dementia (fatal and non-fatal) was extracted from hospital episodes records for incidence and death register for mortality. Incidence follow-up was carried out until the end of March 2021in England and Scotland and the end of March 2018 in Wales. Mortality data were available until February 2021. Nonlinear associations were first investigated using penalised cubic splines fitted in the Cox proportional hazard models. In addition, using MVPA, five categories were created. Associations of these categories with the outcomes were investigated using Cox proportional hazard models. Analyses were adjusted for sociodemographic, lifestyle and health-related factors.

**Results:**

After a median follow-up of 6.3 years, 678 individuals were diagnosed with dementia. Evidence of nonlinearity was observed for all PA modes and all-cause dementia. For categories of MVPA, there was a significant trend towards a low risk of overall dementia when higher levels of MVPA were achieved (HR_trend_ 0.66 [95% CI 0.62 to 0.70]. The lowest risk was identified in individuals who performed more than 1200 MET/min/week, those who had 84% (95% CI 0.12 to 0.21) lower risk of incident dementia compared to those who performed < 300 MET/min/week.

**Conclusions:**

Participants with higher PA levels had a lower risk of incident dementia than those less active, independently of sociodemographic, lifestyle factors and comorbidity. Considering that the majority of previous studies have reported this association using self-reported data, our findings highlight the strong inverse association between PA objectively measured and incident dementia.

**Supplementary Information:**

The online version contains supplementary material available at 10.1186/s12916-021-02172-5.

## Background

Physical activity (PA) is widely acknowledged as associated with several health benefits and reduces the risk of adverse health outcomes throughout the life cycle [1, 2]. Indeed, the World Health Organisation (WHO) Guidelines on Physical Activity and Sedentary Behaviour, published in 2020, have highlighted that ‘each step counts ’[2]. According to these guidelines, adults should engage in at least 150–300 min per week of moderate PA (or 75–150 min per week of vigorous PA or their equivalent to at least 600 metabolic equivalent tasks [MET]/min per week of moderate-to-vigorous PA [MVPA]) [2]. Nevertheless, estimates suggested that 27.5% of the worldwide population was physically inactive in 2016 [3]. In the UK, 25% of individuals older than 16 years were considered physically inactive in 2018 [4].

Evidence suggests PA as a important modifiable risk factor for dementia, with higher levels of PA considered potentially protective against the disease. In fact, the latest dementia expert report, published in 2020 [5], identified 12 major risk factors associated with dementia which are collectively attributable to 40% of dementia cases, among which PA is a key factor. This is highly relevant considering that around 50 million individuals are living with dementia worldwide, at a cost to the global economy of US$1trillion each year [5].

However, to date, research evidence contributing to the understanding of the association of PA with dementia risk has been largely derived from studies using subjective and self-reported data on PA [6–14], which are prone to recall biases, particularly in distinguishing level and intensities of activity [15, 16]. The latter could obscure the true nature and magnitude of the association with dementia. This has been illustrated for other health outcomes, where the health benefits associated with device-measured PA were more than twice as big as those estimated from questionnaires [17]. To date, only one study of 761 older individuals, followed over 3.5 years, has examined the association of device-measured PA with Alzheimer’s disease (AD) [18]. Therefore, robust data on the association of objective measures of PA with dementia and its subtypes remains lacking. Besides, there is no evidence on the dose-response association between the device-measured PA and dementia outcomes, which would be useful to inform recommendations to prevent dementia. Considering the current literature gap, this study aimed to explore the associations of device-measured PA with the risk of dementia incidence and common subtypes (AD and vascular dementia) using the UK Biobank study, the largest prospective cohort with device-measured PA available to date.

## Methods

### Study design and population

The UK Biobank cohort study enrolled over 500,000 participants aged 37–73 years at baseline from the general population (5.5% response rate) [19]. In brief, between 2006 and 2010, participants attended one of 22 assessment research centres across Scotland, England and Wales [20, 21]. All participants completed a touch-screen questionnaire, had physical measurements taken, and provided blood, urine and saliva samples at baseline. The current study includes only a subset of 103,682 participants invited to participate in the device-measured PA study between 2013 and 2015. Of these, 84,854 had data available for PA, dementia outcomes and covariates in this study.

UK Biobank was approved by the North West Multi-Centre Research Ethics Committee (Ref: 11/NW/0382). The study protocol is available online (http://www.ukbiobank.ac.uk/). This work was conducted under the UK Biobank application number 7155. More information about the UK Biobank protocol can be found online (http://www.ukbiobank.ac.uk).

### Device-measured physical activity

**Fig. 1:**
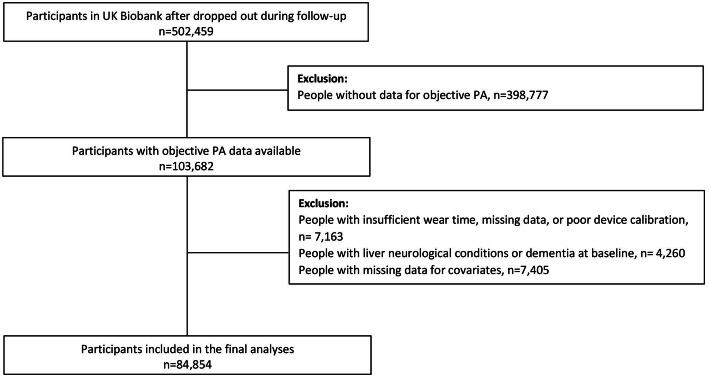
Flow diagram participants included in the study

Axivity AX3 wrist-worn triaxial accelerometer was used to collect objective PA in 103,682 UK Biobank participants between 2013 and 2015. The dominant wrist of each individual was used over a period of 7 days at 100 Hz, as has been described elsewhere [22]. There were 7163 participants with insufficient wear time (< 72 h wear), missing data or poor device calibration who were excluded, leaving 96,519 participants with information regarding PA (Fig. 1). More details about data collection, validation and processing can be found elsewhere [22, 23].

Using the accelerometer data in milligravities (mg) units, we first determined the duration of PA in minutes per week (min/week) of light, moderate and vigorous PA as the time spent between 30-mg and 125-mg, 125-mg and 400-mg and above 400-mg of activity intensity for light, moderate and vigorous PA, respectively [24, 25]. Total PA was estimated as the sum of light, moderate and vigorous PA, whilst moderate-to-vigorous equivalent PA was estimated as the sum between moderate PA and vigorous PA * 2. Then, each PA mode was converted to MET (MET/min/week) as follow: light PA * 3.3, moderate PA * 4 and vigorous PA * 8 [26]. Total PA in MET/min/week was estimated as the sum of these three PA modes, whilst MVPA as the sum of moderate and vigorous PA. Light, moderate, vigorous, total and MVPA in MET/min/week were used as continuous exposure to estimate the linear and nonlinear association with the outcomes.

In addition, individuals were classified in five categories according to their MVPA levels and following cut-off points equivalent to those recommended by the current PA recommendation: (i) < 300 MET/min/week (not meeting the current recommendation of ≥ 600 MET/min/week); (ii) between 300 and 599 MET/min/week (not meeting the current recommendation of ≥ 600 MET/min/week); (iii) between 600 and 899 MET/min/week (meeting the current recommendation); (iv) between 900 and 1199 MET/min/week (meeting the current recommendation); and (v) ≥ 1200 MET/min/week (meeting the current recommendation) [2].

### Incident dementia

Incident dementia (fatal and non-fatal) was extracted from hospital episodes records for incidence and death register for mortality. The date of death was obtained from death certificates held by the National Health Service (NHS) Information Centre (England and Wales) and the NHS Central Register Scotland (Scotland). Dates and causes of hospital admissions were identified via record linkage to Health Episode Statistics (HES) (England and Wales) and the Scottish Morbidity Records (SMR01) (Scotland). Details of the linkage procedure can be found at http://content.digital.nhs.uk/services. The start of follow-up was the date where all device-measured PA measurements were completed. Participants with dementia or any neurological condition prior to that date were excluded from the analysis. Hospital admissions data were available until the end of March 2021 in England and Scotland and theend of March 2018 in Wales. Mortality data were available until the end of February 2021. Therefore, follow-up was censored at these dates.

Using the International Classification of Diseases, 10th revision (ICD-10) [27], AD and vascular dementia were defined as an ICD-10: G30 (Alzheimer’s disease) and F01 (vascular dementia). All-cause dementia (Hereafter ‘dementia’) was defined as F00 (dementia in Alzheimer disease), F01, F02 (dementia in other diseases) or F03 (unspecified dementia) and G30.

### Covariates

Age, when PA data was collected, was determined from dates of birth and PA assessment. Deprivation (area-based socioeconomic status) was resulting from the postcode of residence, using the Townsend score [28]. Ethnicity was self-reported and categorised into white and others (South Asian, black, Chinese, and mixed ethnic background). Educational attainment was based on a self-report of the highest level of qualification and coded as an ordinal variable. Self-reported smoking status was categorised as never, former or current smoker. Height and body weight were measured by trained nurses during the initial assessment. Body mass index (BMI) was calculated as (weight in kg)/(height in m)^2^ and the WHO criteria were applied to categorise participants into underweight < 18.5 kg/m^2^, normal weight 18.5–24.9 kg/m^2^, overweight 25.0–29.9 kg/m^2^ and obese ≥ 30.0 kg/m^2^ [29]. The frequency of alcohol intake was also self-reported at baseline and categorised into five categories: daily/almost daily, 3–4 times a week, once/twice a week, 1–3 times a month, special occasions only and never. The intake of fruit and vegetable, total fish (both oily and non-oily fish), processed meat and red meat was also self-reported and then categorised into ‘do not meet the recommendations’ or ‘meet the recommendations’ following current UK Guidelines as it has been described elsewhere [30]. Prevalent morbidity was ascertained during a nurse-led interview at baseline. We calculated morbidity count based on 43 long-term conditions (including depression) developed initially for a large epidemiological study in Scotland and subsequently adapted for UK Biobank [31, 32]. The complete list of these diseases can be found in the additional file 1. Pairs matching test was used to assess participants’ visual memory (hereafter ‘visual memory’). Participants were asked to memorise the positions of six card pairs and then match them from memory whilst making as few errors as possible. The reaction-time test (timed test of symbol matching) was completed through a touch-screen test (Snap) in milliseconds across trials that contained matching pairs. Due to the low score of these variables, both visual memory and reaction time were transformed to a logarithm scale before including them in the analyses.

### Statistical analyses

Descriptive characteristics by categories of MVPA are presented as means with standard deviation (SD) for quantitative variables and as frequencies and percentages for categorical variables.

Nonlinear associations between the exposures and incident dementia (including AD and vascular dementia) were investigated using penalised cubic splines fitted in Cox proportional hazard models. The penalised spline is a variation of the basis spline, which is not as sensitive to knot numbers and placements as in restricted cubic splines [33]. For these splines, values were truncated to less than 5% and greater than 95% of the values per each PA exposure. After truncation, the minimum value of each exposure was used as the reference group: 5531.904 MET/min/week for total PA, 4424.112 MET/min/week for light PA, 645.12 MET/min/week for moderate PA, 0 MET/min/week for vigorous PA, and 645.12 MET/min/week for MVPA. The proportional hazard assumption was checked using Schoenfeld residuals (the proportional hazard assumptions were all non-significant with a *p* value > 0.05). Follow-up time was used as the time-dependent variable. Individuals with dementia or any neurological condition at baseline (*n* = 4260) were excluded from the analyses.

Associations between categories of MVPA in and incident dementia outcomes were also investigated using Cox proportional hazard models. Individuals in the lowest category (< 300 MET/min/week) were used as the reference group. The results are reported as hazard ratios (HR) and their 95% confidence intervals (95% CIs) as well as absolute risk. The cumulative crude hazard rate between MVPA categories and incident dementia outcomes by follow-up time and age was estimated using the Nelson-Aalen estimator. The rate advancement periods (RAPs)—i.e. the number of additional chronologic years that would be required to yield the equivalent risk rate for dementia incidence among the categories of MVPA—was also estimated as described previously [34, 35]. To calculate RAPs, we divided the logarithm coefficient (HR) for the incidence for the MVPA categories referent to people who did not meet the recommendations for the incidence associated with each yearly increase in age, e.g. $\frac{\log \left(H{R}_{MVPA}\right)}{\log \left(H{R}_{Age}\right)}$.

All analyses were adjusted for a wide range of confounder factors, including age, sex, deprivation, ethnicity (white vs others), education, morbidity count, BMI (continuous), smoking, alcohol intake, fruit and vegetable, total fish, processed meat and red meat intake, log reaction time and log visual memory. Individuals with incomplete data (*n* = 7405) were removed from the analyses. Furthermore, three sensitivity analyses were performed: (i) for the categories of MVPA in MET/min/week excluding people with cardiovascular disease (CVD, heart attack, heart failure, and/or stroke), hypertension and diabetes at baseline (*n* = 21,385); (ii) as per sensitivity 1, but additionally excluding people with any morbidity at baseline (*n* = 29,626); and (iii) for the splines using a 2-year landmark analysis to reduce reverse causality.

R 3.6.1 (using the packages ‘forestplot’, ‘survival’, and ‘spline’) and Stata 17 statistical software (StataCorp LP) were used to perform the analyses. A *p* value below 0.05 was considered statistically significant.

## Results

After excluding people with missing data for the accelerometer measures, covariates and dementia or neurological conditions at baseline, 84,854 individuals were included in this prospective study (Fig. 1). The cohort characteristics by MVPA categories are presented in Table 1. Overall, individuals with higher MVPA levels were younger, less likely to be from deprived socioeconomic status, overweight or obese, current smokers and to have more than one morbidity than those less active (< 300 MET/min/week of MVPA). They were also more likely to be women, to have higher educational attainment, to consume alcohol 3–4 times a week and to follow the recommendations of fruit and vegetables, processed meat and red meat (Table 1).

**Table 1:** Characteristics of the study population by the MVPA categories at baseline

	Not meeting the recommendations		Meeting the recommendations		
	< 300 MET/min/week	300–599 MET/min/week	600–899 MET/min/week	900–1199 MET/min/week	≥ 1200 MET/min/week
**Socio-demographics**					
Total *n* (%)	727 (0.9)	2351 (2.8)	5438 (6.4)	7073 (8.3)	69,265 (81.6)
Age at PA assessment (years), mean (SD)	69.0 (5.8)	68.0 (6.0)	66.6 (6.6)	65.3 (7.0)	61.5 (7.8)
Sex (female), *n* (%)	396 (54.5)	1190 (50.6)	2945 (54.2)	3900 (55.1)	38,905 (56.2)
Deprivation, *n* (%)					
Lower	234 (32.1)	843 (35.9)	1955 (36.0)	2618 (37.0)	26,193 (37.8)
Middle	236 (32.5)	768 (32.7)	1904 (35.0)	2574 (36.4)	23,798 (34.4)
Higher	257 (35.4)	740 (31.4)	1579 (29.0)	1881 (26.6)	19,274 (27.8)
Ethnicity, *n* (%)					
White	713 (98.1)	2310 (98.3)	5307 (91.6)	6913 (97.7)	67,010 (96.7)
Others	14 (1.9)	41 (1.7)	131 (2.4)	160 (2.3)	2255 (3.3)
Educational attainment, *n* (%)					
College/university degree	268 (36.9)	819 (34.8)	2034 (37.4)	2726 (38.5)	30,712 (44.4)
A-levels	94 (12.9)	265 (11.3)	677 (12.5)	965 (13.6)	9214 (13.3)
O-levels	148 (20.4)	498 (21.2)	1186 (21.8)	1518 (21.5)	14,153 (20.4)
CSEs	11 (1.5)	70 (2.9)	171 (3.1)	241 (3.4)	2935 (4.2)
NVQ, HND, HNC or equivalent	48 (6.6)	187 (8.0)	346 (6.4)	432 (6.1)	3617 (5.2)
Other professional qualification	49 (6.7)	171 (7.3)	340 (6.2)	429 (6.1)	3369 (4.9)
None of the above	109 (15.0)	341 (14.5)	684 (12.6)	762 (10.8)	5265 (7.6)
**Lifestyle**					
Smoking status, *n* (%)					
Never	316 (43.5)	1103 (46.9)	2786 (51.2)	3841 (54.3)	40,231 (58.1)
Previous	305 (42.0)	966 (41.1)	2125 (39.1)	2700 (38.2)	24,649 (35.6)
Current	106 (14.5)	282 (12.0)	527 (9.7)	532 (7.5)	4385 (6.3)
Alcohol frequency intake, *n* (%)					
Daily or almost daily	164 (22.6)	495 (21.1)	1262 (23.2)	1606 (22.7)	16,219 (23.4)
3–4 times a week	109 (15.0)	457 (19.4)	1149 (21.1)	1714 (24.2)	18,894 (27.3)
Once or twice a week	142 (19.5)	574 (24.4)	1236 (22.7)	1697 (24.0)	17,673 (25.5)
1–3 times a month	114 (15.7)	293 (12.5)	672 (12.4)	822 (11.6)	7218 (10.4)
Special occasions only	120 (16.5)	337 (14.3)	718 (13.2)	790 (11.2)	5831 (8.4)
Never	78 (10.7)	195 (8.3)	401 (7.4)	444 (6.3)	3430 (5.0)
Fruit and vegetables, *n* (%)					
Meet the recommendations	374 (51.4)	1171 (49.8)	2746 (50.5)	3684 (52.1)	38,389 (55.4)
Total fish intake, *n* (%)					
Meet the recommendations	396 (54.5)	1241 (52.8)	2873 (52.8)	3679 (52.0)	35,152 (50.8)
Processed meat, *n* (%)					
Meet the recommendations	481 (66.2)	1553 (66.1)	3611 (66.4)	4859 (68.7)	49,715 (71.8)
Red meat, *n* (%)					
Meet the recommendations	331 (45.5)	1115 (47.4)	2566 (47.2)	3455 (48.8)	36,479 (52.7)
**Health status**					
BMI (kg/m^2^), mean (SD)	30.2 (6.0)	29.9 (5.8)	28.9 (5.3)	28.1 (5.3)	26.2 (4.2)
BMI categories, *n* (%)					406 (0.6)
Underweight (< 18.5 kg/m^2^)	6 (0.8)	7 (0.3)	15 (0.3)	30 (0.4)	29,206 (42.2)
Normal weight (18.5–24.9 kg/m^2^)	121 (16.6)	416 (17.7)	1247 (22.9)	1851 (26.2)	28,557 (41.2)
Overweight (25.0 to 29.9 kg/m^2^)	262 (36.0)	919 (39.1)	2270 (41.7)	3065 (43.3)	11,096 (16.0)
Obese (≥ 30.0 kg/m^2^)	338 (46.6)	1009 (42.9)	1906 (35.1)	2127 (30.1)	
Reaction time (seconds) (log scale)	6.6 (0.2)	6.6 (0.3)	6.6 (0.2)	6.6 (0.2)	6.5 (0.2)
Visual memory (log scale)	2.4 (0.7)	2.3 (0.7)	2.3 (0.7)	2.3 (0.7)	2.3 (0.7)
Morbidity count, *n* (%)					
None	85 (11.7)	418 (17.8)	1355 (24.9)	2072 (29.3)	30,034 (43.4)
1	188 (25.9)	694 (29.5)	1793 (33.0)	2365 (33.4)	23,278 (33.6)
2–3	347 (47.7)	962 (40.9)	1926 (35.4)	2239 (31.7)	14,429 (20.8)
≥ 4	107 (14.7)	277 (11.8)	364 (6.7)	397 (5.6)	1524 (2.2)

*n*, number; SD, standard deviation; PA, physical activity; CSE, certificate of secondary education; NVQ, national vocational qualification; HND, higher national diploma; HNC, higher national certificate; MET, metabolic equivalent tasks

UK Biobank was approved by the North West

**Fig. 2:**
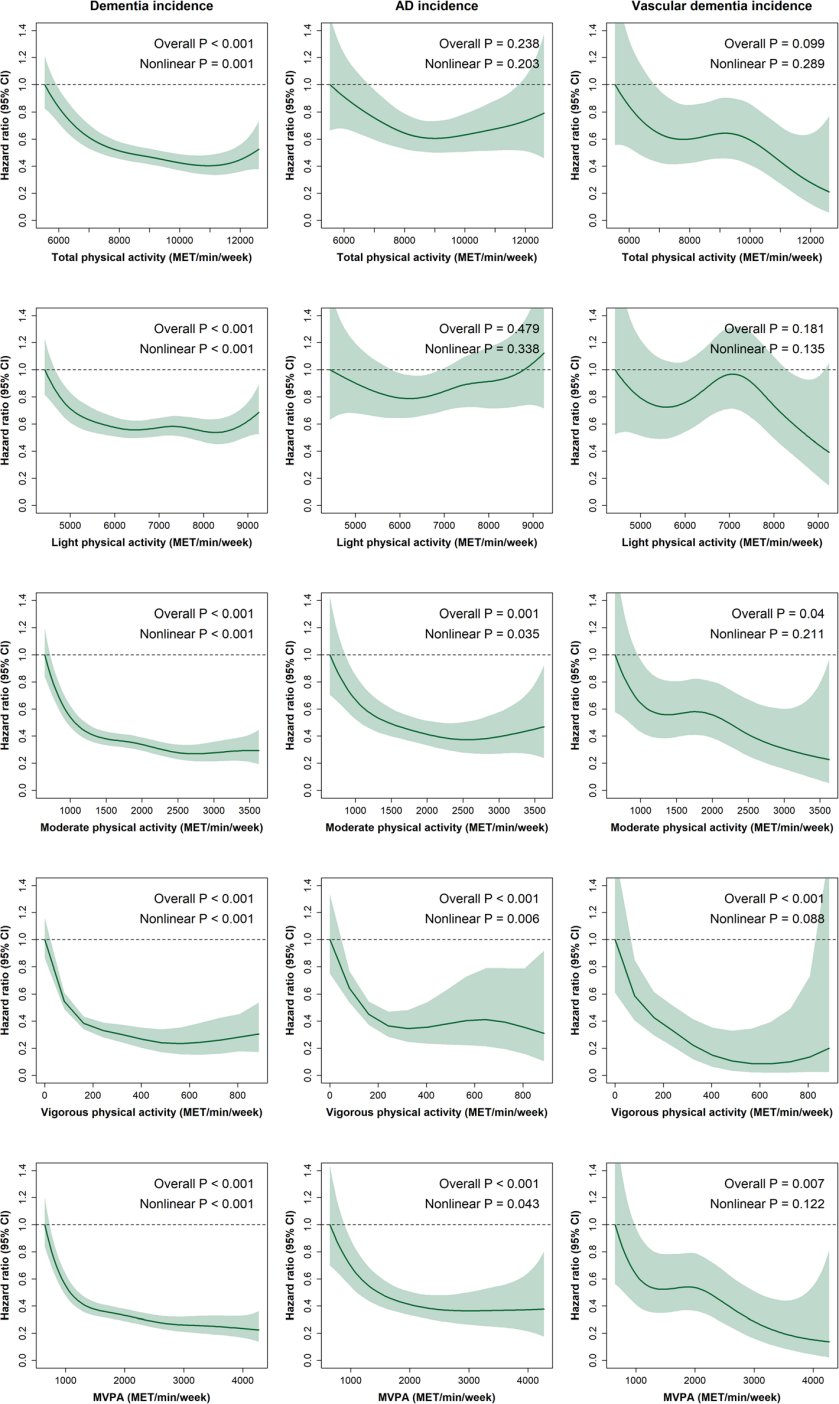
Device-measured PA modes (duration and intensity) and its association with incident dementia (all-cause, Alzheimer’s disease, and vascular dementia). All analyses were performed excluding participants with all-cause dementia and neurological disorders at baseline. Analyses were adjusted by age, sex, deprivation, ethnicity, education, morbidity count, BMI, smoking, alcohol intake, fruit and vegetable, total fish, red meat, and processed meat intake, log reaction time and log visual memory. The minimum value of each exposure was used as a reference group: 5531.904 MET/min/week for total PA, 4424.112 MET/min/week for light PA, 645.12 MET/min/week for moderate PA, 0 MET/min/week for vigorous PA and 645.12 MET/min/week for MVPA. AD, Alzheimer’s disease; MET, metabolic equivalent tasks; PA, physical activity; MVPA, moderate-to-vigorous physical activity

The median follow-up time was 6.3 years (interquartile range 5.8 to 6.8 years). Over this period, 678 (0.8%) individuals were diagnosed with incident dementia. Figure 2 shows the dose-response association between PA domains and dementia incidence (including AD and vascular dementia). Compared to those less active, those who performed between 7000 and 8000 MET/min/week of total PA had ~ 50% lower risk of dementia incidence; even though the risk of dementia decreased up to 60% in those performing between 10,000 and 11,000 MET/min/week of total PA, no further benefits were observed over this point. In terms of light PA, those on the upper end of light PA equivalent to ~ 6000 MET/min/week had ~ 40% lower risk of dementia incidence with similar estimates beyond this point. For moderate PA and MVPA, 1000 MET/min/week was associated with a ~ 60% lower risk of dementia incidence compared to the least active. The risk for dementia was up to a ~ 70% lower on those doing 2500 MET/min/week of moderate PA or MVPA, with no further benefits observed beyond this point (Fig. 2). For vigorous PA, the risk of dementia incidence was between 60 and 70% lower on those individuals doing between 200 and 400 MET/min/week. The association was slightly flat after 400 MET/min/week. Moreover, there was clear evidence of nonlinearity across all PA domains and all-cause dementia (*P* nonlinear < 0.001). Except for light PA, similar risk estimates to those reported for dementia were observed for AD and vascular dementia but with wider confidence intervals (Fig. 2). When PA domains were expressed as minutes per week, similar associations were observed (Figure S1). Finally, when a 2-year landmark analysis was conducted, similar trends were observed across all PA domains and dementia outcomes (Figure S2).

**Fig. 3:**
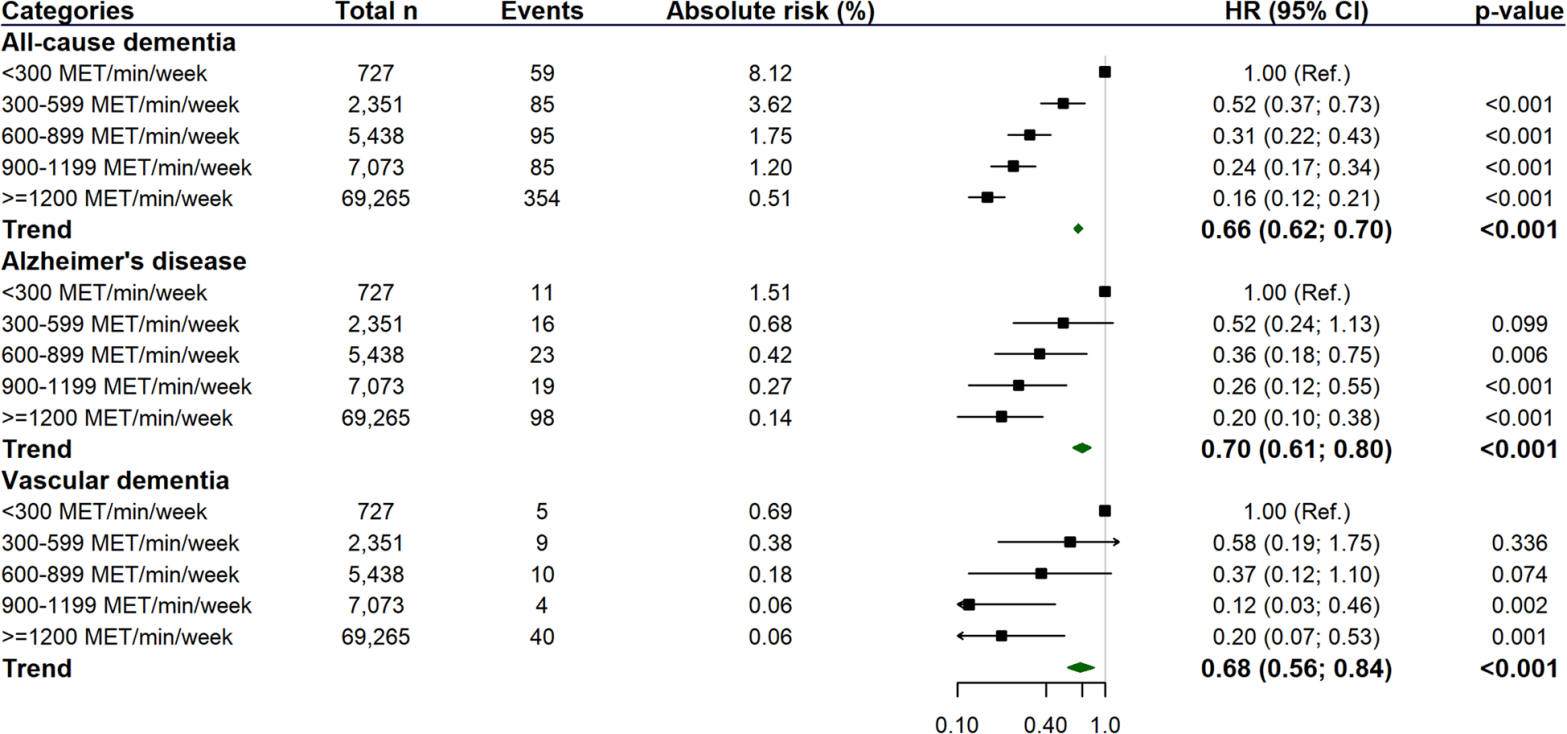
Associations between categories of MVPA (MET/min/week) and incident dementia (all-cause, Alzheimer’s disease, and vascular dementia). All analyses were performed excluding participants with all-cause dementia and neurological disorders at baseline. Analyses were adjusted by age, sex, deprivation, ethnicity, education, morbidity count, BMI, smoking, alcohol intake, fruit and vegetable, total fish, red meat, and processed meat intake, log reaction time and log visual memory. MET, metabolic equivalent tasks; PA, physical activity; MVPA, moderate-to-vigorous physical activity

In terms of categories of MVPA, there was a significant trend towards a low risk of overall dementia (including AD and vascular dementia) when higher levels of MVPA were achieved (Fig. 3). Compared with individuals in the lowest category of MVPA (< 300 MET/min/week), those who performed between 300 and 599 MET/min/week had a 48% lower dementia risk, whilst those who met the guidelines and achieved 600 to 899, 900 to 1199 or ≥ 1200 MET/min/week had a 69%, 76% and 84% lower risk of dementia, respectively (Fig. 3). These associations were slightly attenuated in magnitude but remained significant after excluding participants with CVD, hypertension and diabetes at baseline (sensitivity analysis 1) and also after the exclusion of people with morbidities at baseline (sensitivity analysis 2) (Table S1). In terms of subtypes of dementia, we identified a lower risk of AD in those achieving at least 600 MET/min/week (Fig. 3). These associations remaining significant in sensitivity analysis 1, but were attenuated in sensitivity analysis 2 where only the association between the trend and AD incidence remained significant (HR_trend_ 0.68 [95% CI 0.50 to 0.93]) (Table S1). For vascular dementia, a lower risk was only observed in individuals who performed more than 900 MET/min/week (Fig. 3); however, after excluding people with major diseases and any morbidity at baseline, associations were attenuated, or there was insufficient power to run sensitivity analyses for these outcomes (Table S1).

**Fig. 4:**
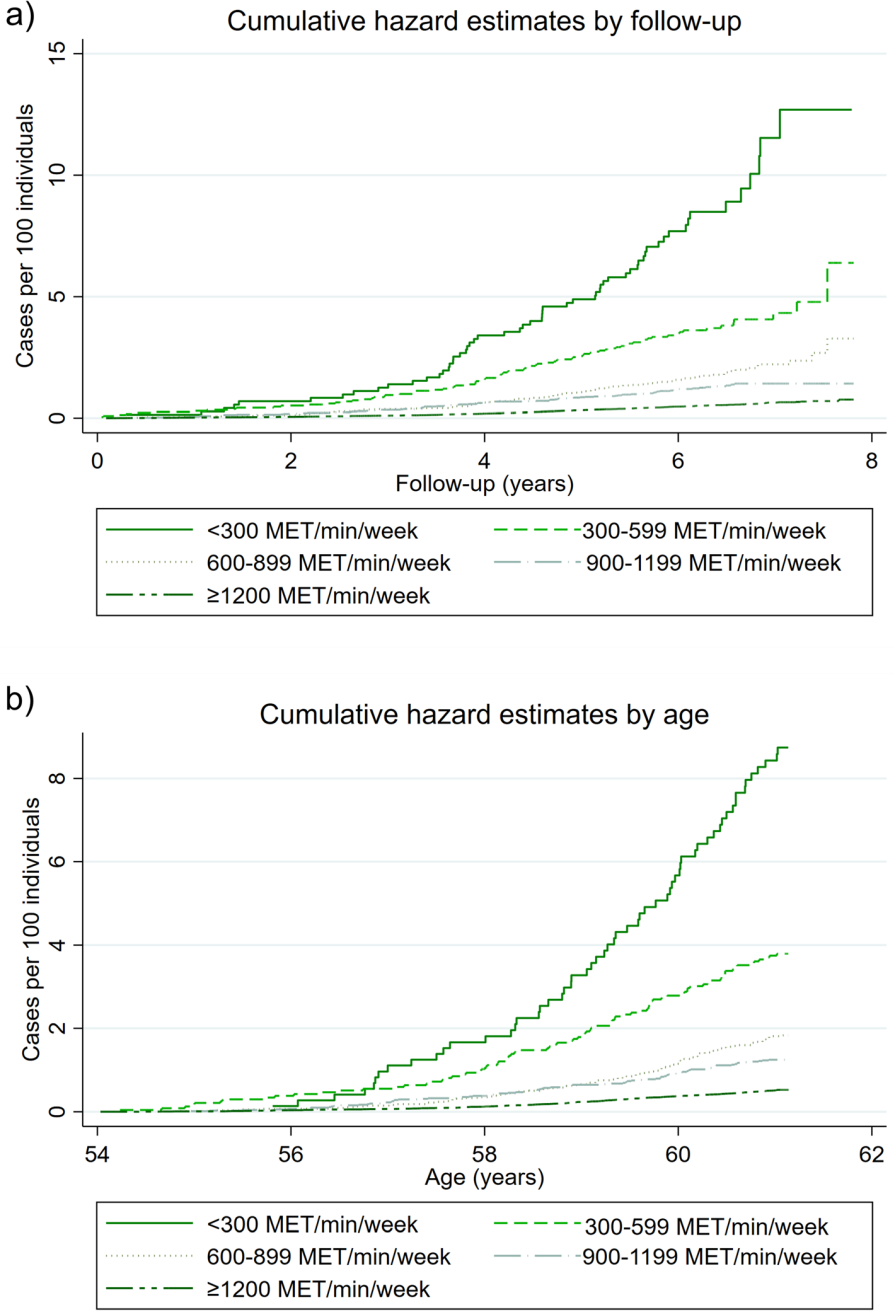
Cumulative hazard plot of all-cause dementia incidence by categories of MVPA, follow-up time and age. Data presented as crude HR by categories of MVPA, follow-up time (**a**) and age (**b**). MET, metabolic equivalent tasks

Besides, when the cumulative crude hazard rate between the categories of MVPA and dementia was determined by time of follow-up and age (Fig. 4), we found that, in comparison to individuals who performed ≥ 300 MET/min/week of MVPA, those who did not have a steeper gradient curve. The crude hazard rate for AD and vascular dementia by follow-up and age are available in Figures S3 to S6.

Finally, based on RAPs analyses (Table 2), the dementia incidence rate estimated for the least active participants (300 MET/min/week of MVPA) was equivalent to those with higher levels of MVPA (300–599; 600–899; 900–1199; and ≥ 1200 MET/min/week), but who were 4.4, 7.9, 9.6 and 12.3 years older, respectively. Except for some exceptions, similar trends were observed for AD and vascular dementia (but with wider CIs, Table 2).

**Table 2:** Rate advancement periods analyses

	RAPs, years (95% CI)				
	Not meeting the recommendations		Meeting the recommendations		
	< 300 MET/min/week	300–599 MET/min/week	600–899 MET/min/week	900–1199 MET/min/week	≥ 1200 MET/min/week
Dementia incidence	0 (Ref.)	− 4.40 (− 7.59; − 2.00)	− 7.89 (− 11.6; − 5.38)	− 9.62 (− 13.5; − 6.87)	− 12.3 (− 16.2; − 9.94)
Alzheimer’s disease incidence	0 (Ref.)	− 3.76 (− 10.2; 0.57)	− 5.87 (− 12.3; − 1.34)	− 7.74 (− 15.2; − 2.78)	− 9.25 (− 16.5; − 4.50)
Vascular dementia incidence	0 (Ref.)	− 4.16 (− 21.6; 2.94)	− 7.59 (− 27.5; 0.50)	− 16.2 (− 45.6; − 4.07)	− 12.3 (− 34.6; − 3.33)

All analyses were performed excluding participants with all-cause dementia and neurological disorders at baseline. Analyses were adjusted by age, sex, deprivation, ethnicity, education, morbidity count, BMI, smoking, alcohol intake, fruit and vegetable, total fish, red meat, and processed meat intake, log reaction time and log visual memory. Individuals who performed < 150 min/week were used as the reference group. MET, metabolic equivalent tasks

## Discussion

Our findings demonstrated a dose-response relationship between device-measured PA and risk of incident dementia, independent of sociodemographic, lifestyle and health-related confounding factors. Similar results were observed when the analyses were restricted to individuals without morbidity, especially for all-cause dementia. When the associations between the categories of MVPA and dementia risk were investigated, a lower dementia risk was observed with higher levels of MVPA. This lower risk was even observed in individuals who performed between 300 and 599 MET/min/week—i.e. still below the physical activity recommendation of more than 600 MET/min/week—who had 48% lower risk of dementia. The latter supports the statement of the WHO that ‘each step count’ and that PA should be encouraged as a protective factor for dementia risk. Furthermore, if we take into account that achieving more than 300 MET/min/week of MVPA may delay dementia between 4.4 and 12 years (for those who performed between 300 and ≥ 1200 MET/min/week), public strategies are needed during the whole life span, but especially in those individuals at higher risk.

Previous prospective studies have reported an inverse association between self-reported PA and dementia (all-cause and subtypes) in individuals from different backgrounds and ages [6–14]. However, most of the studies are based on self-reported physical activity [6–14], which may introduce biases [15, 16]. Even though our findings go in the same direction as self-reported studies, the magnitude of the association was stronger. Therefore, cost-effectiveness analyses based on device-measured PA could be more likely to capture the true burden of disease and, consequently, the benefits of performing different levels and intensities of PA.

To date, only one previous study has investigated this association using device-measured PA [18]. Buchman et al., after a mean follow-up of 3.5 years of 716 older American individuals belonging to the Rush Memory and Aging Project, demonstrated that participants in the lowest category of total PA (10th centile) had a 2.3-fold higher risk of incident AD compared with those in the highest level (90th centile )[18]. Our study extends these findings and provides novel evidence for the association of PA across a range of dementia outcomes (including all-cause and subtypes). Additionally, we investigated the dose-response relationship between PA intensity subdomains and different dementia outcomes, including individuals from middle and older ages, in one of the largest samples with device-measured PA data available up to date. We accept, however, that some estimates for AD and vascular dementia suffered from low power, and hence findings for dementia are most robust.

Although our study provides evidence of an inverse association between device-measured PA and dementia risk, our findings are not entirely consistent with recent causal evidence derived from Mendelian randomisation (MR) studies [36–38] or meta-analyses of randomised controlled trials [39, 40]. Two previous MR studies found that a genetic instrumental PA variable—which reports the association of the genetic instrument with the exposure of interest—was associated with an increased risk of AD with odds ratios > 2 [36, 37. Yet, both MR studies used instrumental genetic variants derived from associations with self-reported PA. These MR studies contradict a recent 2-sample MR analysis conducted in 21,982 patients with AD and 41,944 cognitively normal controls. The authors reported no association between AD and a genetic instrumental PA variable derived from 8 SNPs (Single Nucleotide Polymorphisms) with device-measured vigorous PA [38]. Our findings on AD and device-measured PA are also in contrast with two meta-analyses of intervention studies, which did not show a protective effect of exercise interventions on the risk of AD. However, these meta-analyses, which included more than 36 studies, concluded that most trials had short (6-months) follow-up (only a few had 1- or 2-year follow-up) and that the quality of the evidence was insufficient to conclude the effectiveness of aerobic training or other PA domains for improving cognition or dementia [39, 40]. Therefore, higher-quality trials with clear intervention criteria, larger samples, and long-term follow-up are needed in the future to evaluate the benefits of exercise for AD and other types of dementia.

Whilst evidence regarding PA and long-term brain health outcomes, such as dementia and AD, is conflicting, the evidence relating to intermediate outcomes supports a link between PA and brain outcomes. Previous studies have reported a positive effect of higher PA levels—measured by device-measured—on brain health, including cognitive abilities [41] and brain structure [42]. In Taiwan, higher levels of light PA and MVPA were associated with a 25% (risk ratio [RR]_adjusted_ 0.75 [0.60 to 0.92]) and 15% (RR _adjusted_ 0.85 [0.75 to 0.95)] reduction in cognitive decline [41]. Individuals from the Rush Memory and Aging Project with higher daily PA levels also had larger grey matter volumes (including subcortical and total volume), highlighting the overall positive effect of PA on mental health and brain structure [42].

### Strengths and limitations

UK Biobank is a large, prospective and well-characterised general population cohort of middle-aged and older adults with data available on a wide range of potential confounders. Furthermore, we used objective PA data which reduces misreporting and dilution bias compared to self-reported data [15, 16]. However, this study has some limitations that need to be considered. Firstly, UK Biobank is not representative of the UK population concerning general characteristics, lifestyle and prevalent disease. Consequently, whilst risk estimates can be generalised [43], summary statistics such as prevalence and incidence should not. Secondly, despite including a comprehensive list of confounding factors in the analyses, unmeasured or residual confounding cannot be ruled out in observational studies. Although we could adjust our models for two cognitive test, the UK Biobank study does not have other measurements such as the Mini-Mental State Examination. Therefore, there may be residual confounding due to baseline cognitive ability, which could overestimate the association. Additionally, we did not adjust for apolipoprotein E (*APOE*)-polymorphism, a major risk factor for dementia. However, a previous study conducted using UK Biobank data demonstrated that the association of device-measured PA with cognitive decline measures was not mediated by *APOE* [44]. Fourthly, our sample may not have sufficient power for dementia subtype analyses, particularly for vascular dementia incidence (numbers of events = 68). Therefore, future studies with larger numbers of vascular dementia cases are needed. Fifthly, our primary analyses used data from hospital admission and death records. The latter may include only advanced or severe cases of dementia primarily. Sixthly, we could not distinguish the accelerations patterns between sedentary behaviours and sleep time; therefore, this study did not present the association between sedentary behaviours and the outcomes of interest. Finally, reverse causation is a major issue when the link between PA and dementia is investigated. Recent observational studies have found that when the PA assessment and diagnosis of dementia were ≥ 10 years apart, there was no association between PA and risk of dementia [45, 46]. In particular, an individual-level meta-analysis of 19 studies—including 363,176 participants and 1300 incident dementia cases—found a HR of 1.01(0.89 to 1.13) when comparing physically active with inactive individuals when restricting follow-up time to ≥ 10 years [45]. Likewise, an analysis of 1.1 million women, with 5873 AD cases, found a  HR of 1.03 (0.97 to 1.09) for the comparison of inactive vs active women and AD risk after 15+ years follow-up [46]. Although we tried to reduce the effect of reverse causation by conducting sensitivity analyses where individuals with CVD or chronic diseases at baseline were excluded and conducting a 2-year landmark analysis, we cannot completely rule out the possibility of reverse causation in our study.

## Conclusion

In conclusion, individuals with higher device-measured PA levels, independent of the type of PA, had a lower risk of incident dementia than those less active. Considering that the majority of previous studies have reported this association using self-reported data, our findings highlight the strong inverse dose-response association between PA and incident dementia. For dementia, we also demonstrated that all PA intensities and durations count as they were all associated with a lower risk of all-cause dementia.

Finally, and considering that active individuals might develop dementia almost 12 years later than those less active, public health policies to increase PA levels should be prioritised throughout the life span, especially in those at higher risk of developing dementia.

## Supplementary Information

**Additional file 1:.** List of morbidities. Table S1. Associations between categories of MVPA and incident dementia (all-cause, Alzheimer’s disease, and vascular dementia). Figure S1. Device-measured PA and its association with indecent dementia (all-cause, Alzheimer’s disease, and vascular dementia) using min/week. Figure S2. Device-measured PA and its association with indecent dementia (all-cause, Alzheimer’s disease, and vascular dementia) using a 2-year landmark. Figure S3. Cumulative hazard plot of Alzheimer’s disease incidence by categories of MVPA and follow-up time. Figure S4. Cumulative hazard plot of Alzheimer’s disease incidence by categories of MVPA and age. Figure S5. Cumulative hazard plot of vascular dementia incidence by categories of MVPA and follow-up time. Figure S6. Cumulative hazard plot of vascular dementia incidence by categories of MVPA and age.

## Data Availability

All UK Biobank information is available online on the webpage www.ukbiobank. Data access is available through applications. This research was conducted using the application number 7155.

## Abbreviations

AD: Alzheimer’s disease
BMI: Body mass index
CIs: Confidence intervals
CSE: Certificate of Secondary Education
CVD: Cardiovascular disease
HES: Health Episode Statistics
HND: Higher National Diploma
HNC: Higher National Certificate
HR: Hazard ratio
ICD: International Classification of Disease
MET: Metabolic equivalent tasks
MG: Milligravities
MVPA: Moderate-to-vigorous physical activity
NHS: National Health Service
NVQ: National vocational qualification
PA: Physical activity
Q: Quartile
SD: Standard deviation
SMR01: Scottish Morbidity Records
WHO: World Health Organization
