# Supplementary material for: Dose-response association between device-measured physical activity and incident dementia: a prospective study from UK Biobank
Source: BMC Med. 2021 Dec 2;19:305. doi: 10.1186/s12916-021-02172-5 (PMC8638378; doi:10.1186/s12916-021-02172-5)
Supplement: Supplementary file 1 — Additional file 1:. List of morbidities. Table S1. Associations between categories of MVPA and incident dementia (all-cause, Alzheimer’s disease, and vascular dementia). Figure S1. Device-measured PA and its association with indecent dementia (all-cause, Alzheimer’s disease, and vascular dementia) using min/week. Figure S2. Device-measured PA and its association with indecent dementia (all-cause, Alzheimer’s disease, and vascular dementia) using a 2-year landmark. Figure S3. Cumulative hazard plot of Alzheimer’s disease incidence by categories of MVPA and follow-up time. Figure S4. Cumulative hazard plot of Alzheimer’s disease incidence by categories of MVPA and age. Figure S5. Cumulative hazard plot of vascular dementia incidence by categories of MVPA and follow-up time. Figure S6. Cumulative hazard plot of vascular dementia incidence by categories of MVPA and age. [file 12916_2021_2172_MOESM1_ESM.docx]

**Additional File 1**

**Dose-response association between device-measured physical activity and incident dementia: a prospective study from UK Biobank**

[List of morbidities 2](#_Toc83823783)

[Table S1. Associations between categories of MVPA and incident dementia (all-cause, Alzheimer’s disease, and vascular dementia). 4](#_Toc83823784)

[Figure S1. Device-measured PA and its association with indecent dementia (all-cause, Alzheimer’s disease, and vascular dementia) using min/week. 6](#_Toc83823785)

[Figure S2. Device-measured PA and its association with indecent dementia (all-cause, Alzheimer’s disease, and vascular dementia) using a 2-year landmark 8](#_Toc83823786)

[Figure S3. Cumulative hazard plot of Alzheimer’s disease incidence by categories of MVPA and follow-up time. 9](#_Toc83823787)

[Figure S4. Cumulative hazard plot of Alzheimer’s disease incidence by categories of MVPA and age. 9](#_Toc83823788)

[Figure S5. Cumulative hazard plot of vascular dementia incidence by categories of MVPA and follow-up time. 10](#_Toc83823789)

[Figure S6. Cumulative hazard plot of vascular dementia incidence by categories of MVPA and age. 10](#_Toc83823790)

# List of morbidities

**Count of 43 chronic illnesses including cancer. Some diseases are grouped.**

1. Hypertension
2. Depression
3. Painful condition
4. Asthma
5. CHD
6. Dyspepsia
7. Diabetes
8. Thyroid
9. Inflammation (rheumatoid arthritis/ other inflammation)
10. COPD
11. Anxiety
12. Irritable bowel syndrome
13. Alcohol problems
14. Other psychoactive substance abuse
15. Constipation
16. Stroke
17. Chronic Kidney disease
18. Diverticular disease of intestine
19. Atrial fibrillation
20. Peripheral vascular disease
21. Heart failure
22. Prostate disorders
23. Glaucoma
24. Epilepsy
25. Dementia
26. Schizophrenia
27. Psoriasis or eczema
28. Inflammatory bowel disease
29. Migraine
30. Chronic sinusitis
31. Anorexia or bulimia
32. Bronchiectasis
33. Parkinson’s disease
34. Multiple sclerosis
35. Viral Hepatitis
36. Chronic liver disease
37. Osteoporosis
38. Chronic fatigue syndrome
39. Endometriosis
40. Meniere disease
41. Polycystic ovary syndrome
42. Pernicious anaemia
43. Cancer

# Table S1. Associations between categories of MVPA and incident dementia (all-cause, Alzheimer’s disease, and vascular dementia).

|  | **Total n** | **Events** | **<300 MET/min/week** | **300-599 MET/min/week** | | **600-899 MET/min/week** | | **900-1,199 MET/min/week** | | **≥1,200 MET/min/week** | | **Trend** | |
| --- | --- | --- | --- | --- | --- | --- | --- | --- | --- | --- | --- | --- | --- |
|  |  |  | **HR (95%CI)** | **HR (95% CI)** | **p-value** | **HR (95% CI)** | **p-value** | **HR (95% CI)** | **p-value** | **HR (95% CI)** | **p-value** | **HR (95% CI)** | **p-value** |
| All-cause dementia | 84,854 | 678 | 1.00 (Ref.) | 0.52 (0.37; 0.73) | <0.001 | 0.31 (0.22; 0.43) | <0.001 | 0.24 (0.17; 0.34) | <0.001 | 0.16 (0.12; 0.21) | <0.001 | 0.66 (0.62; 0.70) | <0.001 |
| Sensitivity 1 | 63,469 | 383 | 1.00 (Ref.) | 0.41 (0.24; 0.70) | 0.001 | 0.28 (0.17; 0.45) | <0.001 | 0.23 (0.14; 0.37) | <0.001 | 0.14 (0.09; 0.21) | <0.001 | 0.66 (0.60; 0.72) | <0.001 |
| Sensitivity 2 | 33,843 | 156 | 1.00 (Ref.) | 0.33 (0.12; 0.94) | 0.039 | 0.26 (0.10; 0.67) | 0.005 | 0.20 (0.08; 0.51) | 0.001 | 0.11 (0.04; 0.25) | <0.001 | 0.63 (0.54; 0.73) | <0.001 |
| Alzheimer’s disease | 84,854 | 167 | 1.00 (Ref.) | 0.52 (0.24; 1.13) | 0.099 | 0.36 (0.18; 0.75) | 0.006 | 0.26 (0.12; 0.55) | <0.001 | 0.20 (0.10; 0.38) | <0.001 | 0.70 (0.61; 0.80) | <0.001 |
| Sensitivity 1 | 63,469 | 105 | 1.00 (Ref.) | 0.30 (0.09; 0.99) | 0.049 | 0.35 (0.13; 0.90) | 0.030 | 0.28 (0.10; 0.73) | 0.009 | 0.15 (0.06; 0.37) | <0.001 | 0.68 (0.58; 0.81) | <0.001 |
| Sensitivity 2 | 33,843 | 41 | 1.00 (Ref.) | 0.24 (0.01; 4.02) | 0.323 | 0.64 (0.08; 5.39) | 0.679 | 0.28 (0.03; 2.67) | 0.271 | 0.19 (0.02; 1.53) | 0.120 | 0.68 (0.50; 0.93) | 0.015 |
| Vascular dementia | 84,854 | 68 | 1.00 (Ref.) | 0.58 (0.19; 1.75) | 0.336 | 0.37 (0.12; 1.10) | 0.074 | 0.12 (0.03; 0.46) | 0.002 | 0.20 (0.07; 0.53) | 0.001 | 0.68 (0.56; 0.84) | <0.001 |
| Sensitivity 1 | 63,469 | 28 | 1.00 (Ref.) | 0.39 (0.02; 6.40) | 0.511 | 0.89 (0.10; 7.69) | 0.917 | 0.13 (0.01; 2.15) | 0.154 | 0.34 (0.04; 2.74) | 0.313 | 0.77 (0.54; 1.09) | 0.140 |
| Sensitivity 2 | 33,843 | 10 | 1.00 (Ref.) | *- | *- | *- | *- | *- | *- | *- | *- | *- | *- |

All analyses were performed excluding participants with all-cause dementia and neurological disorders at baseline. The main model was adjusted by age, sex, deprivation, ethnicity, education, morbidity count, BMI, smoking, alcohol intake, fruit & vegetable, total fish, red meat, and processed meat intake, log reaction time and log visual memory. Sensitivity 1: as per the main model but excluding people with CVD, hypertension and diabetes at baseline. Sensitivity 2: as per sensitivity 1 but also excluding people with morbidity count at baseline. Alzheimer’s disease and vascular dementia incidence was estimated using the main model. Individuals who performed <300 MET/min/week were used as the reference group. MET: metabolic equivalent tasks; PA: physical activity; MVPA: moderate to vigorous physical activity. *- Not sufficient data for sensitivity analysis.


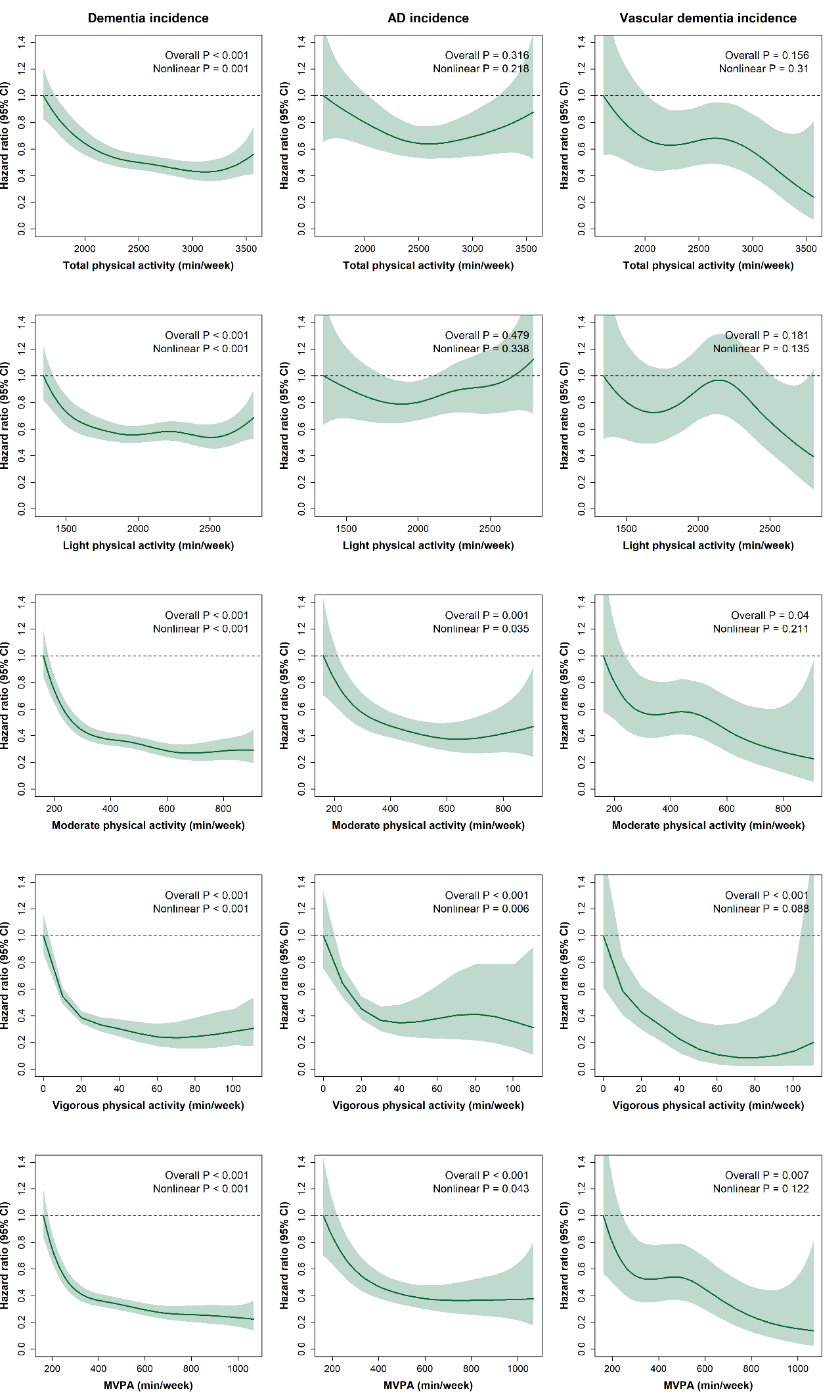


# Figure S1. Device-measured PA and its association with indecent dementia (all-cause, Alzheimer’s disease, and vascular dementia) using min/week.

All analyses were performed excluding participants with all-cause dementia and neurological disorders at baseline. Analyses were adjusted by age, sex, deprivation, ethnicity, education, morbidity count, BMI, smoking, alcohol intake, fruit & vegetable, total fish, red meat, and processed meat intake, log reaction time, log visual memory, fruit & vegetables, fish, red meat and proceed meat intake. The minimum value of each exposure was used as a reference group: 1,340.64 min/week for light PA, 161.28 min/week for moderate PA, 0 for vigorous PA, 161.28 for MVPA and 1,612.8 min/week for total PA. AD: Alzheimer’s disease; PA: physical activity; MVPA: moderate to vigorous physical activity.


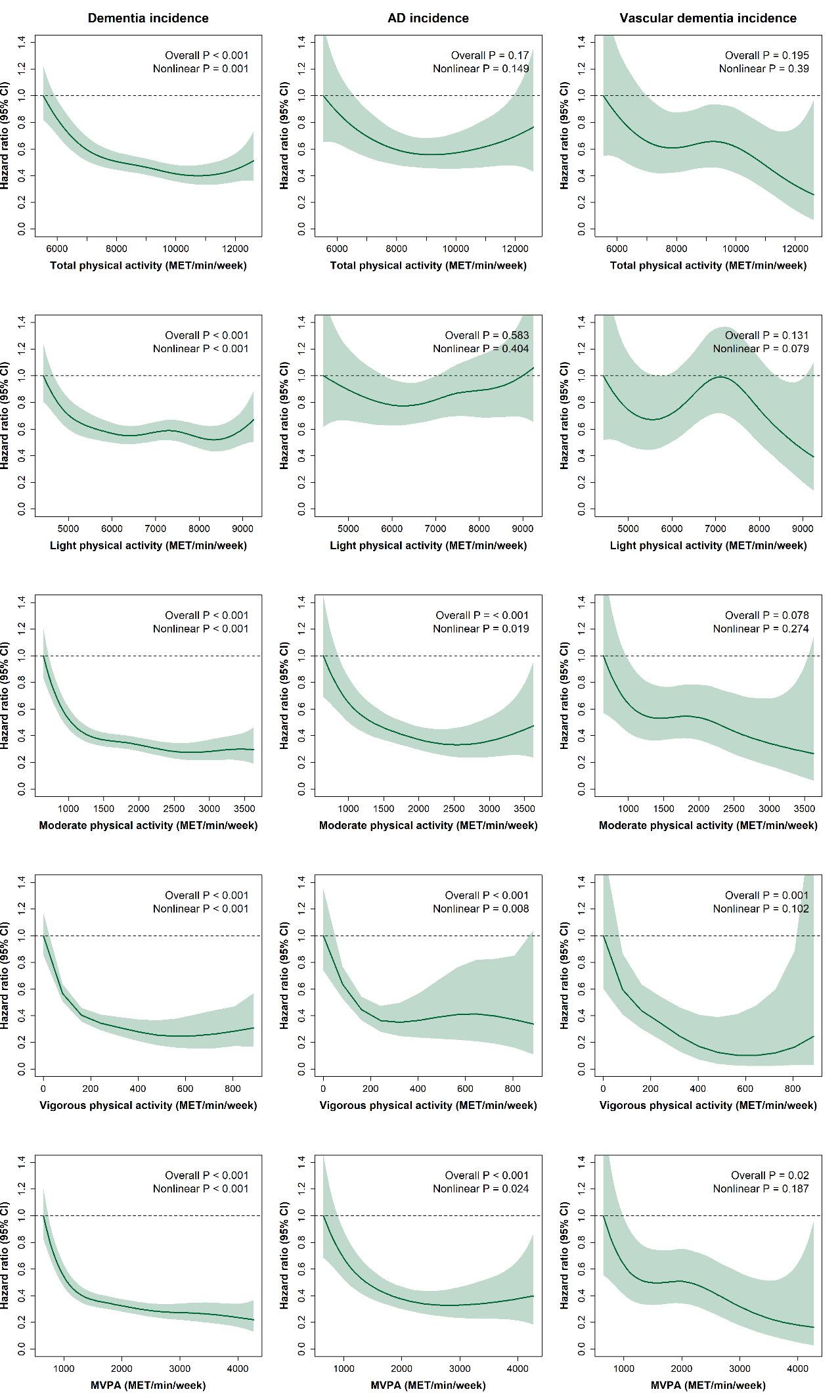


# Figure S2. Device-measured PA and its association with indecent dementia (all-cause, Alzheimer’s disease, and vascular dementia) using a 2-year landmark

All analyses were performed excluding participants with all-cause dementia and neurological disorders at baseline using a 2-year landmark. Analyses were adjusted by age, sex, deprivation, ethnicity, education, morbidity count, BMI, smoking, alcohol intake, fruit & vegetable, total fish, red meat, and processed meat intake, log reaction time, log visual memory, fruit & vegetables, fish, red meat and proceed meat intake. The minimum value of each exposure was used as a reference group: 5,531.904 MET/min/week for total PA, 4,424.112 MET/min/week for light PA, 645.12 MET/min/week for moderate PA, 0 MET/min/week for vigorous PA, and 645.12 MET/min/week for MVPA. AD: Alzheimer’s disease; PA: physical activity; MVPA: moderate to vigorous physical activity.


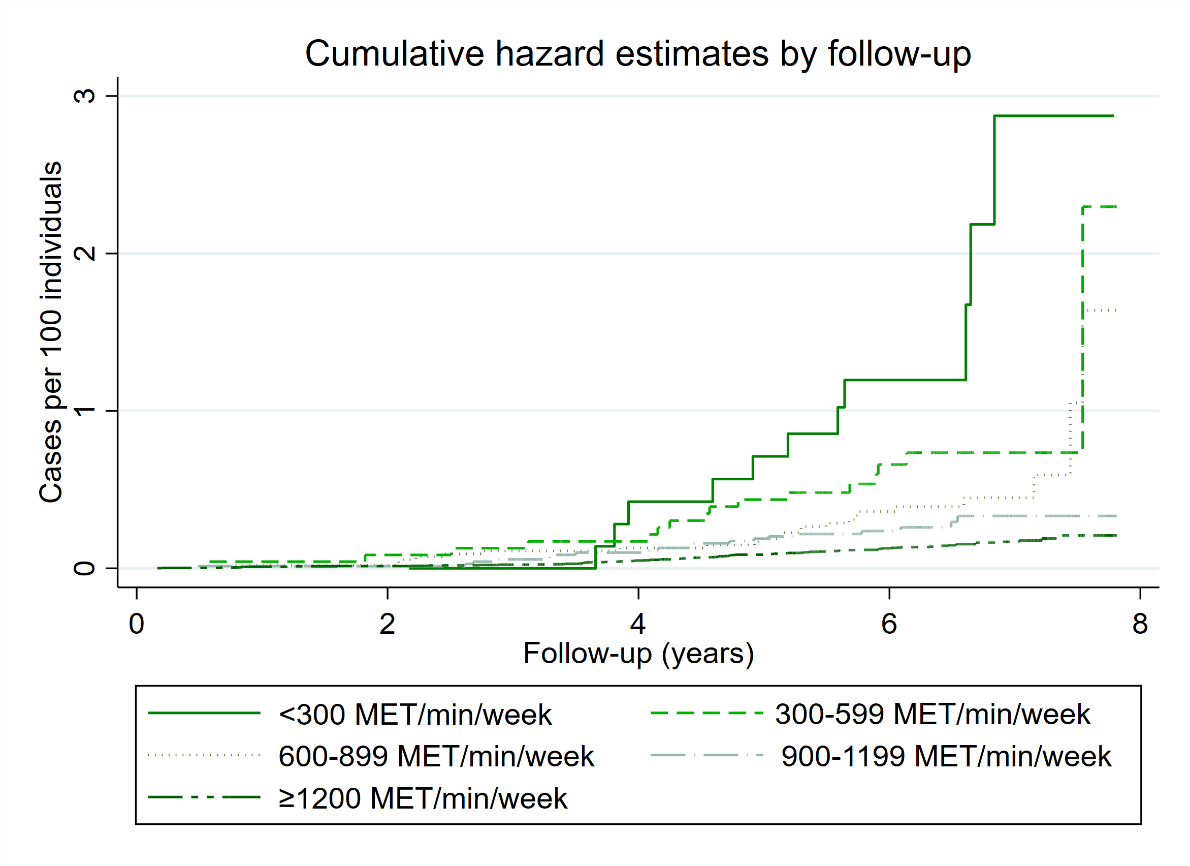


# Figure S3. Cumulative hazard plot of Alzheimer’s disease incidence by categories of MVPA and follow-up time.

Data presented as crude HR by categories of MVPA.

**
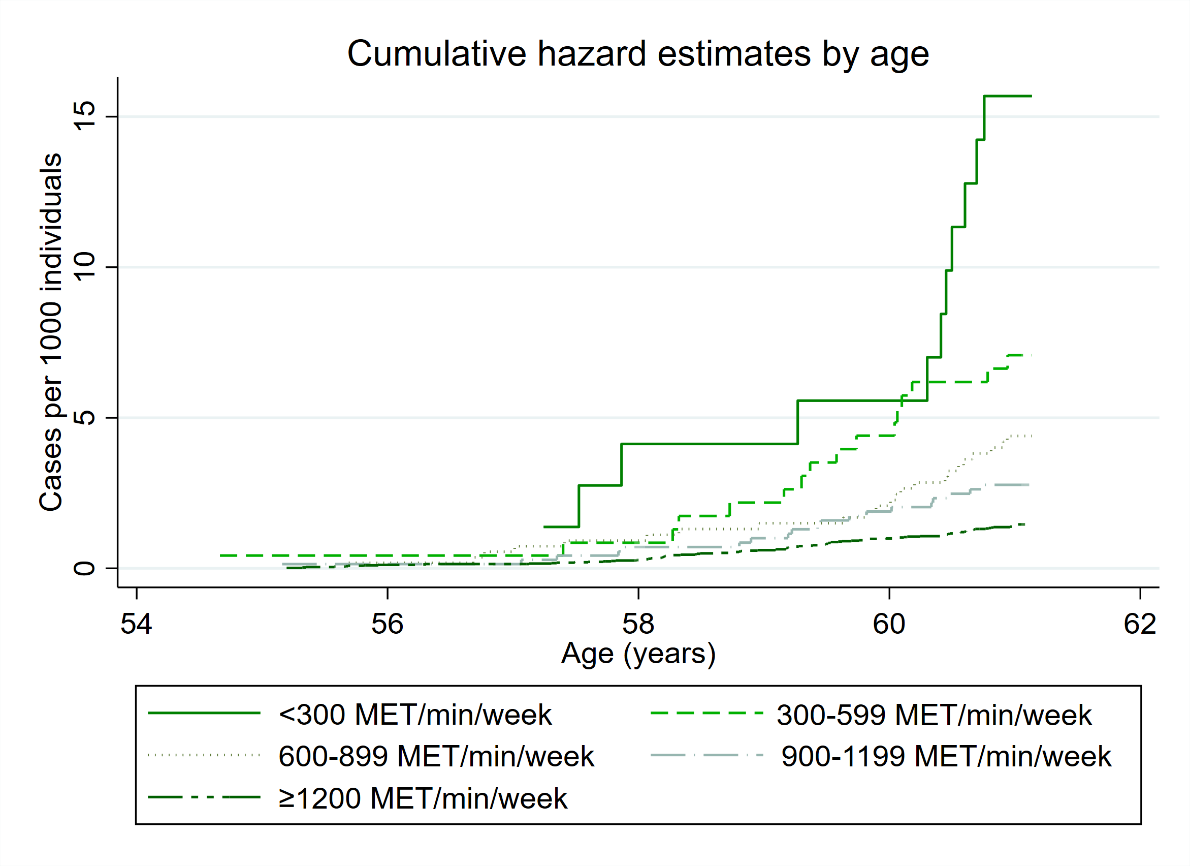
**

# Figure S4. Cumulative hazard plot of Alzheimer’s disease incidence by categories of MVPA and age.

Data presented as crude HR by categories of MVPA.


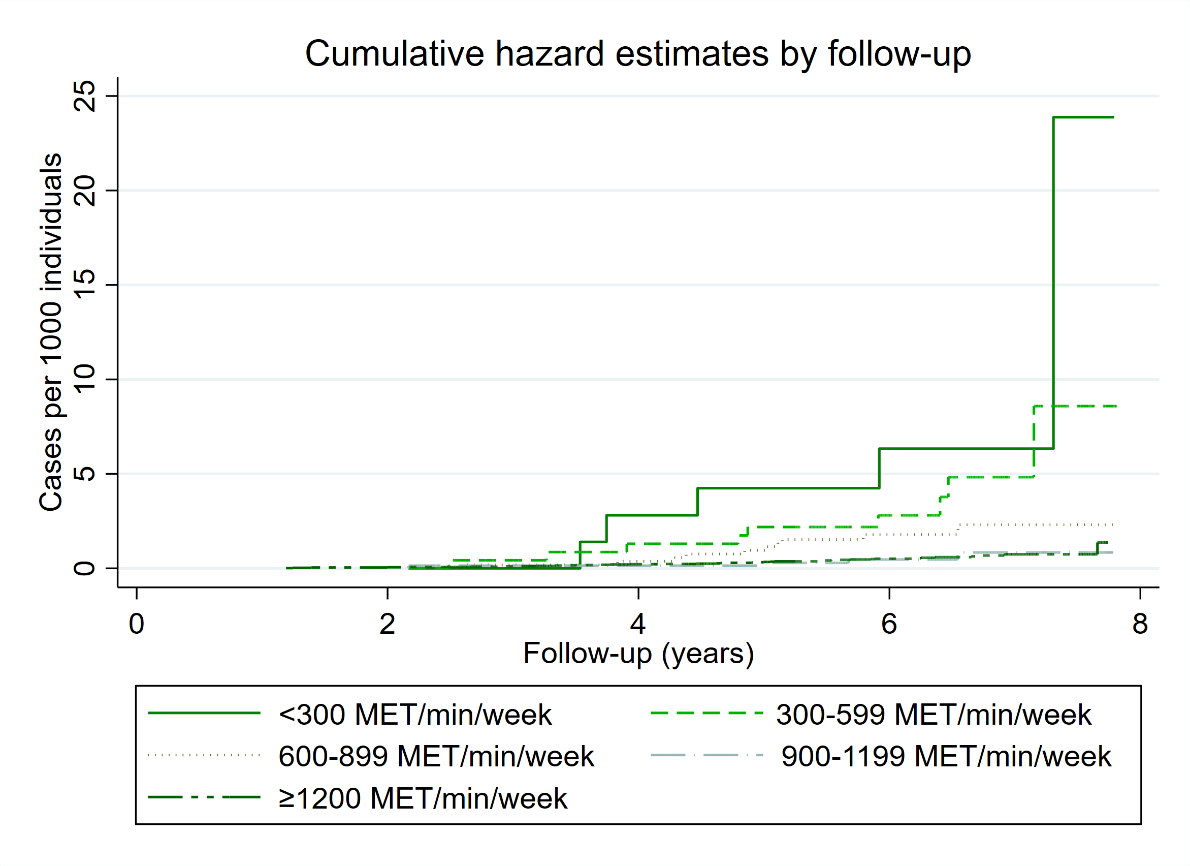


# Figure S5. Cumulative hazard plot of vascular dementia incidence by categories of MVPA and follow-up time.

Data presented as crude HR by categories of MVPA.

**
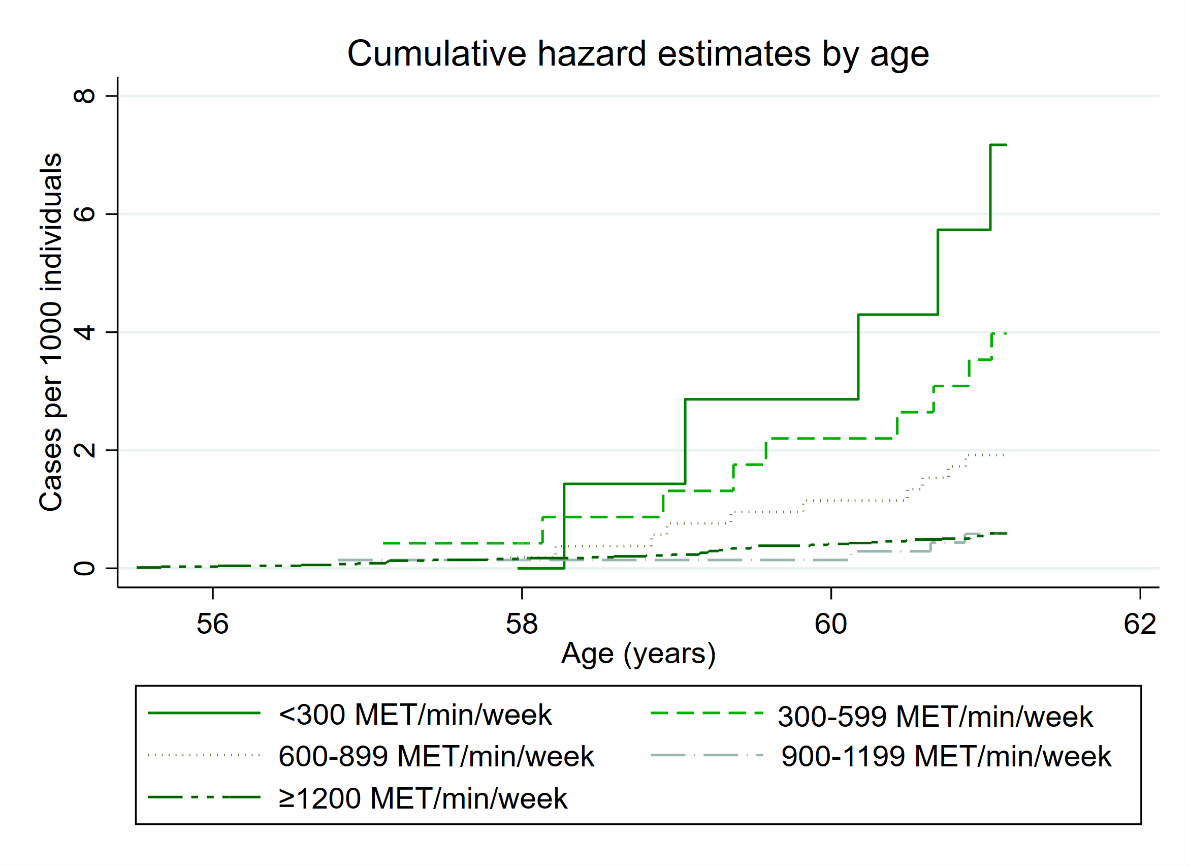
**

# Figure S6. Cumulative hazard plot of vascular dementia incidence by categories of MVPA and age.

Data presented as crude HR by categories of MVPA.
